# Oral Tongue Squamous Cell Carcinoma in Young Women: A Matched Comparison—Do Outcomes Justify Treatment Intensity?

**DOI:** 10.1155/2014/529395

**Published:** 2014-03-10

**Authors:** Ryan P. Goepfert, Eric J. Kezirian, Steven J. Wang

**Affiliations:** Department of Otolaryngology-Head and Neck Surgery, University of California, San Francisco, 2233 Post Street, San Francisco, CA 94115, USA

## Abstract

*Background*. The incidence of oral tongue squamous cell carcinoma (OTSCC) in young women is increasing with uncertain outcomes compared to traditional patients. Published outcomes data are at odds in this cohort of young women. *Methods*. Retrospective analysis comparing demographic, clinicopathologic, and outcomes data of women OTSCC patients younger than 45 years old matched 1 : 2 by stage with men both younger and older than 45 and women older than 45. *Results*. No disease-free or overall survival differences were found between cohorts. Young women were significantly more likely to receive radiation therapy, particularly in stage I disease, even when controlling for common pathologic indications. *Conclusions*. OTSCC in young women was not associated with worse outcomes compared to a matched cohort of other patients. Increased frequency of radiation treatment for this cohort may not be justified.

## 1. Introduction

Oral tongue squamous cell carcinoma (OTSCC) is the most common tumor in the oral cavity with an estimated 12,770 new cases in the United States in 2012 [1]. According to a 2011 published analysis of SEER data, the overall incidence of OTSCC was stable from 1975 to 2007 but was increasing in women and more specifically increasing in the subset of young, white women [2]. Other publications have documented similar findings suggesting that there are gender differences in OTSCC incidence, pathogenesis, and outcomes [3, 4]. Venables and Craft (1967) and Byers (1975) discussed some of the unique disease patterns among tongue cancers in young, white women including possibly more aggressive tumor behavior and delays in initial diagnosis due to low clinical suspicion [5, 6]. Unfortunately, many of the same themes and uncertainties persist several decades later. Attempts to compare clinicopathologic characteristics and outcomes data of young patients with OTSCC to a more traditional patient cohort of older patients have yielded disparate results. Given that this discrepancy in reported outcomes influences treatment discussions and patient recommendations at our institutional level, we sought to review our own patients with OTSCC and analyze for differences among different cohorts. The aims of this study were to compare young women with OTSCC to a matched cohort of young men and older women with respect to (1) clinical and histopathologic characteristics, (2) primary surgical and adjuvant therapy regimens, and (3) disease-specific and overall survival.

## 2. Materials and Methods

After approval from the Committee on Human Research (CHR 11-05565), a review of the medical records at the University of California, San Francisco, was conducted for all patients with a diagnosis of OTSCC from 1997 to 2011. Patients were selected using the following criteria: consent for access to medical records for research purposes, histopathologic confirmation of squamous cell carcinoma of the oral tongue, primary surgical treatment at our institution, and a minimum follow-up period of two years or until death. Medical records were reviewed and demographic, clinical, histopathologic, and outcome data were transferred to a standardized spreadsheet in a secure format. Patients were excluded if they had a prior history of malignancy of the upper aerodigestive tract or skin of the head and neck, had evidence of distant metastases at presentation, had prior radiation therapy of any site, or had prior resection of the primary lesion. Women aged 45 or younger were identified and matched at a 1 : 2 ratio to men aged 45 or younger and women over age of 45 on the basis of overall pathologic stage. Given the inability to identify sufficient men younger than 45 and women older than 45, additional men older than 45 were included in the matched cohort. The 7th Edition of the American Joint Committee on Cancer (AJCC) TNM staging system was used to stage each patient.

Data were analyzed as categorical or continuous variables, as appropriate, using standard descriptive statistics. Chi-squared tests and Student's *t*-tests evaluated the association between young women and the matched cohort for categorical and continuous variables, respectively. Cox regression models compared survival and freedom from recurrence for young women and the matched cohort, with adjustment for potential confounders. Logistic regression analysis examined the association between undergoing adjuvant therapy and young women versus the matched cohort, also with adjustment for potential confounders. All calculated *P* values were 2-sided, and *P* values less than 0.05 were considered statistically significant. Analyses were performed using Stata Version 10.0 (StataCorp LP, College Station, TX).

## 3. Results

A total of 121 patients met the selection criteria, 18 of which were women under 45 years of age. These patients were matched to a group of 36 patients including 11 men under age of 45, 11 women over age of 45, and 14 men over age of 45, 8 of which were between 45 and 50 years old. Relevant demographic information is listed in Table 1.

The matched cohort was older on average compared to the young women. No differences existed in race, Charlson comorbidity index, alcohol use, stage, or tumor grade. There was no difference in smoking status (current, prior, or never), but there was a trend towards greater tobacco exposure in the matched group smokers compared to the young women smokers (Table 1). Other pertinent specific information includes the following: one young woman and one young man were relatively immunosuppressed on long-term low-dose corticosteroids for autoimmune disorders, two young women were pregnant within 1 year of their cancer diagnosis, and one young man was HIV-positive without AIDS on antiretroviral medications.

All patients underwent resection of primary tumor to varying degrees in concordance with the size of the primary lesion. The resection specifics are listed in Table 2. All patients underwent unilateral or bilateral elective, selective, or modified radical neck dissection depending on preoperative clinical and radiologic staging as well as tumor location. No patients had delayed or salvage neck dissection. No statistically significant differences existed in resection specifics.

The overall AJCC and TNM stages for all patients are listed in Table 3. Analysis of final histopathologic findings revealed no significant differences in depth of invasion, tumor grade, lymphovascular invasion (LVI), perineural invasion (PNI), surrounding dysplasia, extracapsular spread (ECS), total lymph nodes removed, or pathologically positive lymph nodes. Final pathologic analysis of the permanent surgical margins was stratified according to the following rubric with regard to tumor location: negative as greater than 5 mm, close as between 1 and 5 mm, and positive as 1 mm or less. There were no statistical differences in margin status and no patients had frankly positive permanent surgical margins. Histopathologic findings are shown in Table 3.

With regard to adjuvant therapy, 56% (30/54) of the overall group were treated with radiotherapy and 20% (11/54) underwent combined chemoradiotherapy. No patients were treated primarily with either chemotherapy and/or radiation therapy or treated with neoadjuvant therapy. Analysis of adjuvant treatment revealed that the cohort of young women was more likely to undergo radiation therapy as part of their treatment compared to the matched cohort. This was statistically significant for both overall and stage 1 disease (Table 4) and remained statistically significant when controlling for stage, LVI, PNI, margin status, and histologic tumor grade (Table 5). Among the patients with stage 1 disease, 57% (4/7) of young women received adjuvant radiation therapy but none (0/14) in the matched group (*P* = 0.002). All patients with greater than one positive node or ECS received adjuvant radiation or combined therapy.

**Figure 1:**
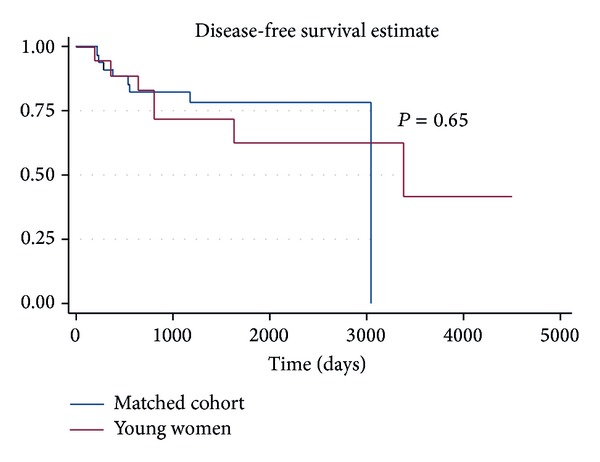
Disease-free survival for young women with OTSCC versus matched cohort.

**Figure 2:**
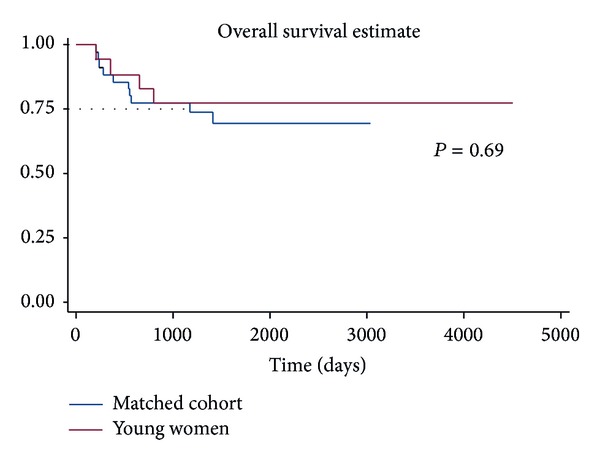
Overall survival for young women with OTSCC versus matched cohort.

Median follow-up among survivors was 64 months (range 26–150 months) in the cohort of young women and 49 months (range 7–100 months) in the matched group. Recurrences occurred in seven of the 18 young women (39%). Five of these patients died of their cancer, while two remain cancer free after salvage surgical resection. Nine of the 36 matched patients recurred (25%), all of which eventually died of their disease. One additional matched patient died from complications of a comorbid condition approximately 7 months after treatment. There were no significant differences with regard to local, regional, or distant pattern of failure between groups. Analysis of outcomes revealed no statistical differences in disease-free survival (DFS) (*P* = 0.65) or overall survival (OS) (*P* = 0.69) between the young women and their matched cohort (Figures 1 and 2). No statistical difference remained in DFS or OS between cases and controls when adjusting for age, overall AJCC stage, and treatment with adjuvant radiation therapy (*P* = 0.36 and 0.36, resp.).

## 4. Discussion

While traditional carcinogens of tobacco and alcohol remain implicated in the majority of cases of OTSCC in older patients, determining a potentially different inciting factor in young patients as well as the contribution of concurrent tobacco and alcohol use in these patients, particularly young women, has been elusive [7–9]. Significant research has focused on this topic including screening for specific mutations of known tumor suppressors, oncogenes, and replication pathways and investigations into the role of viral mediators including human papilloma virus and other novel viral carcinogens [10–14]. Unfortunately, no clear inciting event, pathway, or etiology has been identified.

Appropriate primary and adjuvant treatment for young women with early stage OTSCC remains a topic of great debate and has been the subject of a recently published Phase II clinical trial [15]. These disagreements seem largely due to discrepancies in published outcomes data. A number of historic case series are at odds with regard to outcomes in young patients with OTSCC, and this discordance persists in more recent compilations [5, 6, 16, 17]. Sarkaria and Harari published their institutional series in 1994 including a meta-analysis of all prior published series [18]. Their results indicated higher rates of locoregional failure and higher mortality rates compared to similar series of older adults with OTSCC. A matched analysis by Friedlander et al. found a trend towards poorer prognosis in young patients with OTSCC but failed to achieve statistical significance [19]. A meta-analysis and institutional review by Pitman et al. in 2000 found no outcome differences and only a higher rate of local recurrence compared to a control group of older patients with OTSCC [20]. Popovtzer et al. reported that overall outcomes among young patients with OTSCC were similar to older patients [21]. However, they identified a subset of young patients with a distinctly aggressive clinical course and higher mortality rate. There was no stated association of gender with these outcome differences although women outnumbered men by 3 : 1 in the young cohort. A later publication in 2010 from the same institution indicated that patients younger than 30 with OTSCC presented with advanced tumor stages and had a different failure pattern compared to older patients (distant versus locoregional recurrence, resp.); however, there were no differences in disease-free or overall survival [22]. A matched analysis by Garavello et al. in 2007 found age to be an independent predictor of recurrence (grouped together as local, regional, and distant) in addition to being associated with decreased disease-specific and overall survival [23]. Additional case series published during this period describe equally varied results [24–27].

A review of the preceding published case series reveals several notable differences in their methodology and analysis. These studies often utilized different age thresholds defining “young” (<30 years versus <40 years versus <45 years). Some studies were analyses of unmatched groups of patients, several series spanned many decade time intervals, some studies included tumors from other oral cavity and oropharyngeal sites, and other studies included patients with varied treatment regimens such as primary external beam radiation therapy without neck dissection. Considering these many differences, the lack of consensus on outcomes is not surprising.

Our retrospective study found no clinicopathologic or outcome differences between women aged 45 or younger and a matched cohort of other patients with OTSCC. Our results indicated that young women are significantly more likely to receive adjuvant radiotherapy, particularly in stage I disease, even when adjusting for recognized indications based on primary tumor pathologic characteristics. It is possible that the observed disparity in adjuvant therapy may be responsible in part for the lack of disease outcome differences. Or, this lack of difference may be secondary to a lack of power given the relatively few number of patients in our study. Attempting a scientifically valid regression analysis for all easily measurable factors influencing outcomes was not possible in this series given the small sample size. However, there were no differences in DFS or OS when controlling for age, stage, and use of adjuvant radiation therapy. This discrepancy in interpretation of National Comprehensive Cancer Network (NCCN) guidelines regarding adjuvant therapy in young women with OTSCC has not been reported previously in the literature. Principal among the possible explanations for this difference is clinician bias during treatment recommendation conversations. This bias could easily be explained by the lack of concordance in the literature with regard to outcomes as well as to anecdotal, subjective, or “experience-” based determinations of the aggressiveness of disease. Alternatively, use of adjuvant therapy could be motivated by deeper emotional responses at the personal and/or societal level that accompany a diagnosis of cancer in not only a young person, but also a young woman. The fear and impact of such reality could easily lead to a scenario where clinicians recommend or patients request maximal primary and adjuvant therapy, regardless of stage and recommended indications, in an effort to attain the best possible chance of cure.

The significant difference in use of adjuvant radiation therapy raises an important question; namely, is this cohort of young women being unnecessarily subjected to the long-term side-effects of high-dose adjuvant radiation therapy in pursuit of treatment of a disease for which the literature cannot demonstrate a consistent decrement in outcomes? A recent publication by Thomas et al. on long-term quality of life in young patients treated for squamous cell carcinoma of the oral cavity found that tumor stage and use of radiotherapy were correlated with swallowing outcomes, while only use of radiotherapy was significantly associated with adverse quality of life [28]. Prior reports also suggest that women, young patients, and those treated with radiation therapy tend to report more functional complaints. Since quality of life outcomes can deteriorate over time and other well-known complications of primary and adjuvant therapy may take many years to manifest, only long-term follow-up can provide the answer [29, 30].

There are several important limitations to our study. This analysis was retrospective in nature with shortcomings inherent to this study design. The study was limited by small sample size. In addition, our analysis did not explore differences in primary surgical provider or surgical specialty, location of adjuvant therapy (university versus community-based), and other factors that may impact treatment recommendations and possibly influence outcomes. Lastly, our pathologic analysis did not include analysis for human papilloma virus, although several studies have documented the lack of this virus as an implicating factor in the cohort of young adults with OTSCC [2, 12].

In summary, our study found that young women with OTSCC fared no worse in their outcomes than a matched group of young men, older men, and older women. Young women with OTSCC were more likely to undergo adjuvant radiation therapy, often receiving radiation therapy in situations not supported by NCCN guidelines. Our findings raise the possibility that these patients may have been overtreated. Alternatively, these patients might have had worse outcome without more liberal use of radiation therapy. A multi-institutional study with standardized treatment protocols would help better define the appropriate treatment for this small but increasing group of patients with OTSCC.

## Figures and Tables

**Table 1:** Demographic and clinicopathologic information.

Variable	Young women (*n* of 18), *n* (%)	Matched cohort (*n* of 36), *n* (%)	*P* value
Mean age, years (95% CI)	37.3 (34.3–40.3)	47.9 (43.7–52.0)	0.0010
Number of women/number of men	18/0 (100/0)	11/25 (31/69)	
Charlson comorbidity Index (CCI)			0.22
CCI ≤ 2	15 (83)	23 (64)	
CCI = 3	3 (17)	9 (25)	
CCI > 3	0 (0)	4 (11)	
Smoking history			0.79
Current	3 (17)	9 (25)	
Former	6 (33)	11 (31)	
Never	9 (50)	16 (44)	
Pack-years (mean)	13.1	24.5	0.08
Alcohol abuse history			0.69
Former/current	3 (17)	6 (17)	
Never	15 (83)	30 (83)	

**Table 2:** Primary treatment specifics.

Treatment	Young women (*n* of 18), *n* (%)	Matched cohort (*n* of 36), *n* (%)	*P*-value
Glossectomy type			0.34
Partial	10 (56)	25 (69)	
Hemi	4 (22)	8 (22)	
Subtotal/total	4 (22)	3 (8)	
Neck dissection type			0.70
Unilateral	16 (89)	29 (81)	
Bilateral	2 (11)	7 (19)	
Adjuvant radiotherapy	14 (78)	15 (42)	0.02
Adjuvant chemotherapy	5 (28)	6 (17)	0.33

**Table 3:** Stage and histopathologic data.

Variable	Young women (*n* of 18), *n* (%)	Matched cohort (*n* of 36), *n* (%)	*P* value
Tumor stage (T)			0.22
T1	10 (56)	19 (53)	
T2	3 (17)	10 (28)	
T3	4 (22)	2 (6)	
T4	1 (6)	5 (14)	
Nodal stage (N)			0.72
N0	11 (61)	20 (56)	
N1	1 (6)	6 (17)	
N2*a*/*b*/*c*	0/5/1 (0/28/6)	0/8/2 (0/22/6)	
N3	0 (0)	0 (0)	
Metastases	0 (0)	0 (0)	n/a
Overall AJCC stage			0.99
I	7 (39)	14 (39)	
II	2 (11)	5 (14)	
III	2 (11)	4 (11)	
IV	7 (39)	13 (36)	
Grade			0.70
Well differentiated	6 (33)	11 (31)	
Moderately differentiated	7 (39)	18 (50)	
Poorly differentiated	5 (28)	7 (19)	
Average depth, mm (95% CI)	13.0 (8.5–17.6)	13.5 (9.8–17.2)	0.87
Lymphovascular invasion (LVI)			0.50
Present	4 (22)	4 (11)	
Absent	14 (78)	32 (89)	
Perineural invasion (PNI)			0.51
Present	9 (53)	14 (39)	
Absent	8 (43)	22 (61)	
Final permanent margin			0.57
Negative (>5 mm)	7 (39)	18 (53)	
Close (>1 mm, <5 mm)	6 (33)	10 (29)	
Positive (<1 mm)	5 (28)	6 (18)	
Surrounding dysplasia			0.75
Present	7 (39)	10 (30)	
Absent	11 (61)	23 (70)	
Mean total nodes (95% CI)	42 (29–54)	32 (26–39)	0.09
Mean total positive nodes (95% CI)	1.2 (0.2–2.3)	1.1 (0.6–1.6)	0.83
Extracapsular spread (ECS)			0.89
Present	3 (17)	5 (14)	
Absent	15 (83)	31 (86)	

**Table 4:** Ratio of radiotherapy by AJCC overall stage.

Stage	Young women	Matched cohort	*P* value
I	4/7 (57)	0/14 (0)	0.002
II	2/2 (100)	2/5 (20)	0.15
III	1/2 (50)	2/4 (50)	1.00
IV	7/7 (100)	12/13 (92)	0.45

**Table 5:** Use of adjuvant radiation controlling for common indications.

Category	Odds ratio	*Z* score	*P* value	95% CI
Young women versus matched cohort	33.9	2.05	0.040	1.18–975
Stage I versus stage II	28.5	1.89	0.059	0.88–919
Stage II versus stage III	13.8	1.48	0.138	0.43–440
Stage III versus stage IV	360	3.14	0.002	9.12–14174
LVI, present versus absent	31.1	1.66	0.096	0.54–1788
PNI, present versus absent	1.27	0.21	0.832	0.14–11.9
Margins, negative versus close	4.8	1.11	0.267	0.30–77
Margins, close versus positive	7.3	1.26	0.207	0.33–162
Histologic grade, well versus moderate	1.31	0.82	0.710	0.12–14.1
Histologic grade, moderate versus poor	27.3	1.28	0.201	0.17–4311
